# Environmental enrichment, sexual dimorphism, and brain size in sticklebacks

**DOI:** 10.1002/ece3.2717

**Published:** 2017-02-12

**Authors:** Elisavet A. Toli, Kristina Noreikiene, Jacquelin DeFaveri, Juha Merilä

**Affiliations:** ^1^ Molecular Ecology & Conservation Genetics LabDepartment of Biological Applications & TechnologyUniversity of IoanninaIoanninaGreece; ^2^ Ecological Genetics Research UnitDepartment of BiosciencesUniversity of HelsinkiHelsinkiFinland

**Keywords:** brain size, environmental enrichment, fish, phenotypic plasticity, sexual dimorphism

## Abstract

Evidence for phenotypic plasticity in brain size and the size of different brain parts is widespread, but experimental investigations into this effect remain scarce and are usually conducted using individuals from a single population. As the costs and benefits of plasticity may differ among populations, the extent of brain plasticity may also differ from one population to another. In a common garden experiment conducted with three‐spined sticklebacks (*Gasterosteus aculeatus*) originating from four different populations, we investigated whether environmental enrichment (aquaria provided with structural complexity) caused an increase in the brain size or size of different brain parts compared to controls (bare aquaria). We found no evidence for a positive effect of environmental enrichment on brain size or size of different brain parts in either of the sexes in any of the populations. However, in all populations, males had larger brains than females, and the degree of sexual size dimorphism (SSD) in relative brain size ranged from 5.1 to 11.6% across the populations. Evidence was also found for genetically based differences in relative brain size among populations, as well as for plasticity in the size of different brain parts, as evidenced by consistent size differences among replicate blocks that differed in their temperature.

## 1 Introduction

Given the importance of the central nervous system to organismal performance, and thereby also fitness, the size of the brain and its different parts are likely to be traits under strong optimizing selection. Specifically, increased brain size can enhance individual fitness through improved cognitive ability (Deaner, Isler, Burkart, & van Schaik, 2007; Gibson, 2002; Kotrschal et al., 2013a,b; Striedter, 2005). Moreover, certain brain regions often show an increase in size that is associated with specific ecological conditions likely to select for this growth (Eifert et al., 2015; Gonzalez‐Voyer & Kolm, 2010; Krebs, Sherry, Healy, Perry, & Vaccarino, 1989; de Winter & Oxnard, 2001). Hence, there appears to be a general consensus that intra‐ and interspecific variation in brain size and size of different brain parts is, at least to some extent, dictated by variation in the strength of positive natural selection acting on them.

However, as maintenance of neural tissue is energetically expensive (Aiello & Wheeler, 1995; Isler & van Schaik, 2006, 2009; Mink, Blumenschine, & Adams, 1981; Nilsson, 1996; Soengas & Aldegunde, 2002), these energetic costs are likely to generate selection pressures opposing increases in brain size (Isler & van Schaik, 2009; Kotrschal et al., 2013a). For instance, the fact that the two sexes commonly differ in brain size and size of different brain parts (e.g., Jacobs, 1996; Kotrschal, Räsänen, Kristjánsson, Senn, & Kolm, 2012; Samuk, Iritani, & Schluter, 2014) is likely to be explainable by sex differences in costs and benefits of maintaining certain sized brain or brain regions. For instance, Nottebohm (1981) demonstrated that the song control nuclei in the telencephalon of canaries (*Serinus canarius*) doubled in size during the breeding season, but only in males. Likewise, given the high energetic costs of maintaining neural tissue, the ability to reduce the size of the brain or a particular brain part through phenotypic plasticity when they are not critical for fitness should be favored by selection. It has been proposed that the sexual dimorphism in the three‐spined stickleback brain might be subject to sex‐specific plasticity such that males increase their brain size during the breeding season in response to the increased cognitive demands imposed by mating, nest‐guarding, and parental demands (Herczeg, Gonda, Balazs, Noreikiene, & Merilä, 2015). In this scenario, such plasticity could be adaptive, as the males would escape the energetic costs of maintaining large brains during the nonbreeding season.

There is also evidence to suggest that various animals are capable of increasing their brain size in response to environmental enrichment (Bennett, Diamond, Krech, & Rosenzweig, 1964; Bennett, Krech, & Rosenzweig, 1964; Bennett, Rosenzweig, & Diamond, 1969; Cummins, Walsh, Budtz‐Olsen, Konstantinos, & Horsfall, 1973; Riege, 1971; Scotto Lomassese et al., 2000; Technau, 1984). However, the evidence for this effect from fish studies is conflicting. While some studies have supported this finding of positive effects of enrichment on brain size (DePasquale, Neuberger, Hirrlinger, & Braithwaite, 2016; Herczeg et al., 2015; Näslund, Aarestrup, Thomassen, & Johnsson, 2012), others have found either negative effects (Kotrschal, Sundström, Brelin, Devlin, & Kolm, 2012; Turschwell and White, 2016) or none at all (Burns, Saravanan, & Rodd, 2009; Kihslinger, Lema, & Nevitt, 2006). This is true not only for overall brain size, but also in the size of certain brain regions such as the cerebellum, olfactory bulb, telencephalon, and optic tectum in which both positive (Herczeg et al., 2015; Kihslinger & Nevitt, 2006; Kotrschal, Rogell, Maklakov, & Kolm, 2012; Näslund et al., 2012) and negative (optic tectum; Herczeg et al., 2015) effects of environmental enrichment have been shown. However, in the cases where positive effects have been found, the effects have been conditional to age (Näslund et al., 2012), sex, and social interactions (Herczeg et al., 2015; Kotrschal, Rogell et al., 2012) or other factors such as stress (DePasquale et al., 2016). Hence, there is a great deal of heterogeneity in the observed responses to environmental enrichment, but there remains little understanding of the causes underlying this heterogeneity.

The main aims of this study were to test (1) whether environmental enrichment leads to increased brain size (or size of different brain parts) in three‐spined sticklebacks (*Gasterosteus aculeatus*), (2) whether the effect of enrichment is similar for the sexes, and (3) whether the effects of enrichment and its interaction with sex are similar across multiple populations. To this end, we conducted a common garden experiment in which fish from four different populations were exposed to either a control (bare aquaria) or enriched (spatial complexity generated with physical structures) treatment over a period of 2 months. Based on the results of an earlier experiment which found that males developed larger brains in enriched tanks as compared to females (Herczeg et al., 2015), we expected to see a similar sex‐specific response to environmental enrichment consistent across the four populations tested. Apart from assessing the treatment effects on brain size and size of different brain parts, we also investigated how enrichment influenced growth (i.e., body size) and condition (i.e., residual mass) of the fish.

## 2 Material and Methods

### 2.1 Fish collecting and husbandry

Fish forming the parental generation were collected from four different localities across the Baltic Sea between 5 and 24 June 2015 (Table 1). Live fish were transported to Helsinki and used to make artificial crosses between 6 June and 5 July 2015. Half of the Sylt and Mariager fjord crosses were done in the field, and fertilized eggs were transported to Helsinki in 50‐ml tubes modified with a mesh bottom for water circulation within a cool (10–14°C) and constantly aerated water bath. Fertilized eggs from 10 full‐sib families from each population were first kept in petri dishes until hatching and then transferred to 500‐ml containers. Larvae were fed twice a day with live *Artemia salina* nauplii. After 7 days, the families were transferred to 1.2‐L tanks in Allentown zebrafish racks (Allentown, San Diego, CA, USA), where they continued to be fed *Artemia* naupalii ad libitum for 1–2 months. Each family was then divided into two separate 5‐L tanks on the zebrafish racks, each housing 20 fish. Finely chopped (frozen) chironomid larvae were introduced into their diet by mixing with *Artemia* for 1 month, after which the *Artemia* were eliminated and only whole chironomid larvae were fed. Once the fish were 12–16 weeks old, four fish from each of the 10 families per population (i.e., a total of 160 fish) were randomly chosen to be used in the experiment (see below), which was conducted in a separate room.

**Table 1:** Descriptive information about study populations and samples. Age (in weeks) gives average of the individuals in each of the populations at time of brain measurements

Country	Location	Sea area	Coordinates	Age salinity (ppt)		*n*_Females_	*n*_Males_	*n*_Total_
Germany	Sylt	North Sea	55°01′N, 08°25′E	26	28	21	16	37
Denmark	Mariager	Kattegat	56°38′N, 09°57′E	25	20	15	21	36
Finland	Kotka	Baltic Sea	60°33′N, 27°12′E	23	6	8	21	29
Finland	Oulu	Baltic Sea	65°07′N, 25°14′E	22	3	18	19	37
Total						62	77	139

*N*, sample size for brain measurements.

### 2.2 Experiments

**Figure 1:**
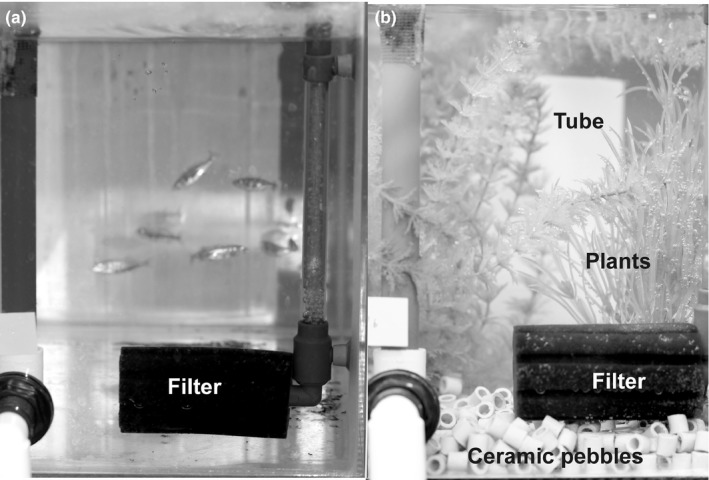
Frontal views of aquaria used in (a) control and (b) enriched treatments

The experiments were conducted in a room fitted with sixteen 38‐L aquaria (L:40 cm × H:30 cm × W:24 cm) distributed among two shelves on two separate sides of the room (henceforth: blocks). Each aquaria was assigned to one of two treatments, “enriched” or “control”. In the enriched treatment, the bottom of the aquaria was covered with ceramic pebbles, along with two artificial plants and a plastic cylinder (23 cm in height, 9 cm in diameter) to generate structural complexity. In the control treatment, the aquaria were left empty, except for the aerator, which was present in both treatments (Figure 1). In order to control for possible aquaria and block effects, two replicate tanks per population and treatment were used (i.e., four populations × two treatments × two replicates = 16 aquaria). Each of the replicates was placed on opposite sides of the room, such that each population and treatment were represented (next to each other) on the same shelf in both blocks. White plastic sheets were placed between aquaria to serve as visual partitions.

The experiments started by introducing one fish from each of the 10 full‐sib families per population into a particular treatment aquaria. Hence, each aquaria housed 10 unrelated fish from a given population. Fish were kept under constant light to mimic summer conditions at the northern latitudes and fed with frozen chironomid larvae (ad libitum) twice daily.

All fish were raised in freshwater, which was maintained at about 17°C (±1°C). However, due to temperature stratification in the experimental room, fish in the two blocks experienced slightly different temperatures (A block: *x* = 17.5°C; max–min = 17.2–17.9°C; B block: *x* = 16.5°C; max–min = 16.4–16.9°C).

Experiments were terminated when the fish were between 22 (Kotka and Oulu) and 26 (Sylt and Mariager fjord) weeks old by euthanizing the fish with an overdose (250 mg/L) of MS222 (tricaine methane sulphonate). In order to scale the size of the brain/different brain parts with individual variation in body size, the standard length (SL; from the tip of the mouth to the end of the tail base) of all individuals was measured with a digital caliper to the nearest 0.01 mm. Body weight was also recorded to the nearest 0.01 g.

Following measurements, fish were immediately placed in a solution containing 4% paraformaldehyde and 2.5% glutaraldehyde in a phosphate‐buffered saline solution and fixed for 5 days.

### 2.3 Brain measurements and sexing

Brains were dissected under a stereomicroscope by removing the top of the neurocranium and severing the cranial and optic nerves and spinal cord. The caudal section of the medulla was cut between the second and the third vertebrae in each fish in order to standardize the measurements. Hence, the “length” measurement for the medulla did not represent the total length of the entire structure, but rather its length until a standardized cutoff point. Brains were kept in a phosphate‐buffered saline solution until they were photographed with a digital camera from the dorsal, lateral, and ventral sides, from a fixed distance. Width, height, and length of six different brain regions (*viz*. olfactory bulb, optic tectum, telencephalon, cerebellum, dorsal medulla, and hypothalamus) were measured with ImageJ (Abràmoff, Magalhães, & Ram, 2004) using landmarks shown in Appendix S1, following Pollen et al. (2007). For bilateral brain parts, both sides were measured and their average was used in all analyses. These data were then fitted to the ellipsoid models (van Staaden, Huber, Kaufman, & Liem, 1994) to estimate the volume of total brain size and size of the different brain parts. The ellipsoid‐model approach is known to yield reliable estimates of brain and brain part sizes, as verified by comparisons with histology and X‐ray micro‐computed tomography‐based estimates (White & Brown, 2015). Furthermore, the correlation between brain size estimates based on ellipsoid model estimates and actual brain wet‐weights (taken with digital balance to the nearest 0.1 mg) in our data was very high (*r* = .91, *n* = 134, *p* < .001). All dissections, digital image analyses, and measurements were conducted by one person (E.T.), and the volume estimates were highly repeatable (all *R* > .78; *p* < .001), as assessed from two repeated measures (both photography and digital measures were repeated) of 12 individual brains following Becker (1992). Although there was little mortality (1.25%) during the experiments, some brains were damaged during dissections and only 139 brains were available for measurements (Table 1).

As the vast majority of the fish at the end of the experiment were not in breeding condition, sexing by phenotypic criteria was not reliable. Therefore, microsatellite markers were used for sex identification. The details of molecular sexing procedures are given in Noreikiene et al. (2015). In short, sex identification was based on amplifying a part of the 3′UTR of the NADP‐dependent isocitrate dehydrogenase (*Idh*) locus, which yields two bands for male and one band for female three‐spined sticklebacks (Peichel et al., 2004) in the populations used in this study (cf. Toli, Calboli, Shikano, & Merilä, 2016).

### 2.4 Statistical analyses

We used general linear mixed models to analyze variation in brain size and size of different brain parts. In these models, the brain traits were treated as response variables, and population, sex, treatment, and block were added as fixed factors. In order to control for allometric scaling, (log) standard length (qualitatively similar results were obtained using [log] body mass) was added as a covariate in all models. In addition, tank (i.e., individual aquarium unit) was added as random factor to control for nonindependence among individuals in a given tank. To simplify the models, all interactions except the two‐way interaction between sex and treatment were omitted. Significant main effects for the population term were followed by post hoc tests (Tukey's HSD). In addition to investigating variation in brain traits, we also tested how the treatments influenced standard length, body mass, and residual mass (i.e., body condition) of individuals. This was done by fitting linear mixed models similar to those described above, but with (log) standard length added as a covariate in the analysis of body condition.

To verify that lack of treatment effects was not due to a lack of statistical power, we also calculated effect size estimates for the treatment effects using Cohen's *d* (Cohen, 1988). These were calculated from back‐transformed least square estimates (and their confidence intervals) obtained from the models reported in Table 2 assuming *n* = 8 (number of replicate tanks per treatment) in calculations. The latter means that the *d*‐estimates were conservative (i.e., estimated effect sizes were larger than would have been obtained by adopting larger n for calculations).

**Table 2:** Linear model results of brain and body size traits. Tabled values are *F*‐values from linear models for treatment (Tre), population (Pop), sex, block (Blo), standard length (SL), and for sex–treatment interaction. SSD(%) gives the degree of sexual dimorphism (in %), Tre (%) the degree of difference between treatment means as calculated from the back‐transformed least‐square means in the model. For SSD (%), positive values indicate male‐biased SSD, and negative values, female; for Tre (%), positive values indicate larger trait mean in control and negative values indicate larger trait mean in treatment conditions. Tank refers to the proportion of total variance in a given trait explained by the random effect of tank

Trait	Tre	Pop	Sex	T × S	Blo	SL	Tank	SSD(%)	Tre(%)
Brain (wgt)	0.18	5.86*	35.05***	0.45	5.59*	276.72***	0.0	10.2	−0.6
Brain (vol)	0.03	6.95**	22.87***	0.30	2.15	272.86***	6.3	8.0	0.3
*Dorsal medulla*	0.20	3.74*	2.35	0.21	3.35°	101.49***	0.1	5.1	−1.5
*Telencephalon*	0.00	1.92	18.35***	0.12	0.43	64.35***	2.4	11.6	−0.2
*Optic tectum*	0.12	8.41**	9.63**	0.88	4.29°	150.79***	0.0	8.0	−0.9
*Cerebellum*	1.12	1.95	9.91**	0.39	7.04*	129.55***	0.0	9.1	2.7
*Olfactory bulb*	0.00	2.17	0.01	0.00	0.03	8.47**	0.2	0.6	−0.2
*Hypothalamus*	0.10	1.66	8.91**	0.17	0.25	67.82***	25.4	10.3	−2.1
Standard length	0.29	13.56***	19.29***	0.17	4.42°	–	0.0	−6.3	−0.5
Body mass	0.64	2.47	31.07***	0.13	0.00	–	4.0	−28.4	−4.2
Condition	0.53	1.33	17.00***	0.23	1.89	549.35***	19.4	−9.3	−2.7

*p* < .10, *p < .05, **p < .01, ***p < .001.

All analyses were conducted on log(10)‐transformed trait values using software JMP Pro 11 (*ver*. 11.0.0).

## 3 Results

### 3.1 Brain size and size of different brain parts

**Figure 2:**
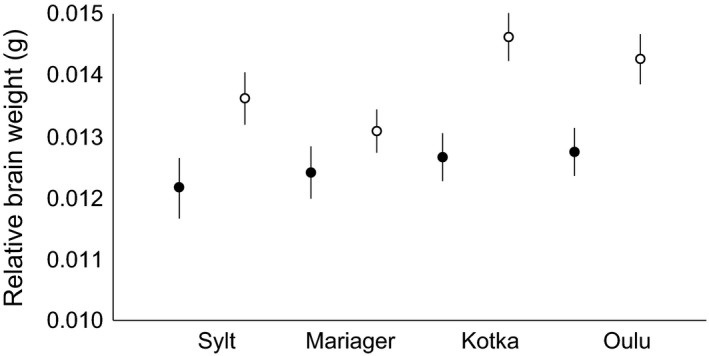
Mean (±SE) brain weight of female (black circles) and male (open circles) sticklebacks in four different populations. The plotted values are least square mean estimates from the model in Table 2. To avoid problems with back transformation of SE values, the model was run without log transformation to obtain the plotted values

Linear models fitted for different measures of total brain volume and volume of different brain parts revealed no main or interaction effects of treatment on any of the brain traits (Table 2). However, for most of the brain traits, significant main effects of population and sex were detected (Table 2). All sexually dimorphic brain traits showed male‐biased sexual size dimorphism (Table 2; Figure 2). The effect of (log) standard length on brain traits was always highly significant, whereas the random effect of tank was appreciable only in the case of the hypothalamus (Table 2). For overall brain size and three brain parts (optic tectum, dorsal medulla, and cerebellum), there were also suggestive and significant block effects (Table 2). These effects owed to the fact that the fish from the A block tended to exhibit larger brains (volume: A: 9.46 ± 0.11; B: 9.19 ± 0.11 mm^3^), optic tectum (A: 5.35 ± 0.079; B: 5.09 ± 0.078 mm^3^), dorsal medulla (A: 0.70 ± 0.01; B: 0.66 ± 0.01 mm^3^), and cerebellum (A: 0.83 ± 0.017; B: 0.77 ± 0.017 mm^3^) than those from the B block.

### 3.2 Body size, mass, and condition

Population and sex differences in body size were significant, with females on average larger than males of the same age (Table 2). The population differences in size arose because individuals from the Mariager population were larger than those from all other populations; differences among the other three populations were nonsignificant (Tukey's HSD, *p* > .05). There were no significant treatment or population effects on body mass and condition, but the significant effect of sex revealed female‐biased dimorphism in all these traits (Table 2). The random effect of tank was appreciable only for body condition (Table 2).

The lack of treatment effects on all studied traits was unlikely to owe to low statistical power of the experiment because the proportional difference in treatment means (Tre%) was most of the time about 10 times smaller than that between the sexes (SSD%; Table 2), and the effect sizes were small (average over all traits <0.20; Appendix S2).

## 4 Discussion

In contrast to our expectations, we did not find any evidence for a positive effect of environmental enrichment on the development of brain size or the size of any brain parts in either of the sexes in any of the four populations tested. The same applied to body size, body mass, and condition of the fish, indicating a lack of positive effect on growth and energy balance of the individuals. Although similar outcomes have been recorded in some earlier studies (reviewed in Näslund & Johnsson, 2016), our findings are noteworthy in light of the results from an earlier study conducted on this species, which found evidence for a positive effect of enrichment on male brain size (Herczeg et al., 2015). In contrast, we found that the degree of sexual size dimorphism (SSD) in brain was similar in both treatments and all populations included in the current study. While the SSD in brain size was slightly higher (on average) than that reported in a Finnish population of this species (Herczeg et al., 2015), it was nevertheless lower than that reported in an Icelandic population of three‐spined sticklebacks (Kotrschal, Räsänen et al., 2012). In the following, we will discuss these findings and their implications for our understanding of environmental enrichment on brain size variation and SSD in the brain in particular.

In contrast to the results of an earlier study (Herczeg et al., 2015), enrichment did not have any effect on brain size. One possible explanation for these contrasting results is that effects are population‐specific, and because the earlier study used a different population, this could account for the discrepancy. However, this seems unlikely because the population used by Herczeg et al. (2015) was also a Baltic Sea population, geographically relatively close (ca. 100 km) to one of the populations used in this study (Kotka). Furthermore, if this geographic variation/population‐specific response to enrichment was the reason for the discrepancy among results between studies, we would have expected to uncover some degree of variation in the responses to enrichment among the four populations included in the current study.

Differences in treatment conditions and/or timing of the treatments could provide another possible explanation for the discordant results among studies. In the current study, the treatments started when the fish were ca. 3 months old and continued over a period of 10 weeks, whereas those in the Herczeg et al. (2015) study were initiated when the fish were already about 5 months old, and continued over a period of 4 weeks. Furthermore, the amount of environmental complexity differed between this and the earlier study: Herczeg et al. (2015) used much larger aquaria with different kinds of physical complexity as compared to that employed in the current study. While it is not clear why these differences would directly influence the outcomes, it remains a logical possibility that they impacted brain development in different ways (see also: Brydges & Braithwaite, 2009), or that the differences in timing or duration of the treatments made the difference. For instance, Näslund et al. (2012) observed that while the effect of environmental enrichment on brain development of Atlantic salmon (*Salmo salar*) was clear in the early stages of development, it dissipated as the fish grew larger. Interestingly, our results in comparison with those of Herczeg et al. (2015) are in contrast to this finding, as they found significant treatment effects at later developmental stages. This could be in part due to the fact that because the males in the Herczeg et al. (2015) study were older and hence closer to sexual maturity, they might have perceived the enrichment as potential breeding habitat, so their response to this enrichment treatment was in fact a reflection of preparation for parental care. Hence, differences in timing, maturation, and details of treatments might have resulted in these differing outcomes.

The lack of treatment effects in our study is particularly interesting in light of the potential for enrichment to indirectly effect social interactions. Namely, although the density of individuals was the same in both treatments, individuals in the enriched treatment had more possibilities of isolating themselves from social interactions with conspecifics than those in control treatments. Indeed, it was noted—especially during feeding—that fish in the control tanks had a much stronger tendency to shoal, whereas those in the enrichment treatment were more independent (E. Toli, personal observation). As social interactions are known (e.g., Gonda, Herczeg, & Merilä, 2009; Technau, 1984) or suspected (e.g., Turschwell and White, 2016) to influence brain development (see Gonda, Herczeg, & Merilä, 2013 for a review), it is possible that reduced frequency of social interactions in the enriched treatment had negative influence on brain development. However, as the density of individuals (ca. 0.2 individuals per liter) in our study was identical to that in Herczeg et al. (2015)—where differences in brain structures were observed between treatments—this suggests that social interactions alone are an unlikely explanation for the difference between this and the earlier study.

Although we did not find any treatment effects, we recorded consistent and pronounced male‐biased SSD in brain size across all four study populations, a finding that is consistent with earlier reports in other stickleback populations (Herczeg, Välimäki, Gonda, & Merilä, 2014; Herczeg et al., 2015; Kotrschal, Räsänen et al., 2012; Samuk et al., 2014). The male‐biased SSD in brain size has been hypothesized to result from selection stemming from the cognitive demands of mate attraction and/or paternal care (Kotrschal, Räsänen et al., 2012). Studying the “white” phenotype of three‐spined sticklebacks, which, in contrast to the “normal” phenotype, does not exhibit paternal care, Samuk et al. (2014) found evidence for reversed SSD in the brain size of white sticklebacks. This leads to a suggestion that the male‐biased SSD in normal sticklebacks is mainly driven by cognitive demands of paternal care (Samuk et al., 2014). As most fish in our experiments were not yet in breeding condition, it is possible that they were not expressing SSD to its maximal extent. That said, the levels of SSD recorded in this study (ca. 5.1–11.6%) were higher than those (ca. 4%) reported by Herczeg et al. (2015), even though the fish used here were younger—hence, likely farther from reproductive condition. To date, the highest report of SSD in stickleback brain size is 23%, which comes from a study of wild‐caught Icelandic stickleback in breeding condition (Kotrschal, Räsänen et al., 2012). Hence, further studies should test whether the outcome of environmental enrichment could be detected at the stage when the fish are actually breeding and exercising paternal care.

Interestingly, some evidence was found to indicate consistent differences in the size of two different brain parts between the replicates/blocks used in this experiment. Fish reared in A block had significantly larger brains and cerebellum and tended to have larger optic tecta and dorsal medulla than those reared in B block. The only systematic difference between A and B blocks we could measure or anticipate was temperature, with fish in the former experiencing about 1°C warmer water temperature than those in latter. Hence, this might suggest that the observed effects on brain development could relate to temperature differences, which are known to have wide‐ranging effects on development of ectothermic animals (Angilletta, Steury, & Sears, 2004). However, it is unclear whether and why slightly warmer temperature should facilitate brain development especially in the view that fish from the two blocks did not differ in body size, mass, or condition suggesting that energetic challenges due to positive effect on temperature on metabolic rates were not at play. Whatever the causal mechanism and significance of the replicate specific differences in brain part sizes, these findings testify to the plasticity in brain size and size of different brain parts.

Finally, we note that laboratory‐based common garden studies—as applied here—represent a fundamentally important approach in evolutionary biology. By allowing environmental sources of variation on trait expression to be controlled for, they allow inferences to be made about genetically based evolutionary transformations. However, common garden situations constitute artificial settings, and may render inferences nonapplicable to situations in the wild. For instance, trait heritabilities measured in the wild and laboratory can be quite different (Weigensberg & Roff, 1996). In the same vein, there is increasing evidence for consistent differences in brain size and size of different brain parts among wild and laboratory‐reared fish, and that these differences are directly attributable to phenotypic plasticity (e.g., Burns et al., 2009; Eifert et al., 2015; Gonda, Herczeg, & Merilä, 2011; Marchetti & Nevitt, 2003; Park, Chase, & Bell, 2012). These studies have found that brain size or size of different brain parts is usually reduced in the fish reared in the laboratory as compared to those caught from the wild. While such responses could be viewed as being adaptive under the environmental enrichment hypothesis (i.e., fish raised in simple laboratory environments reduce their investment in maintaining large brains), it seems equally likely that such changes could also represent stress responses to confinement to unnatural aquarium conditions (e.g., Turschwell & White, 2016). Fish grown in laboratory conditions lack many chemical, physical, and biological stimuli present in the wild and this could directly influence their brain development. As for the results of the present study, the lower levels of male‐biased SSD in this study as compared to that in the Kotrschal, Räsänen et al. (2012) study could be a manifestation of this problem. Further studies comparing levels of SSD in wild‐collected and laboratory‐reared fish from the same populations would be needed to address this possibility.

In conclusion, the results of this study confirm the generally male‐biased SSD in stickleback brain, but find no evidence to suggest that environmental enrichment has positive effects on development of brain size and size of different brain parts in either of the sexes.

## Conflict of Interest

None declared.
