# Supplementary material for: Environmental enrichment, sexual dimorphism, and brain size in sticklebacks
Source: Ecol Evol. 2017 Feb 12;7(6):1691–8. doi: 10.1002/ece3.2717 (PMC5355184; doi:10.1002/ece3.2717)

**Appendix S1.**

Illustration of the measurements taken from the dorsal, lateral and ventral views of brains to determine the size of various brain parts in *G. aculeatus*.W, H and L refer to width, height and length, respectively. 1: telencephalon; 2: optic tectum; 3: cerebellum; 4: dorsal medulla; 5: olfactory bulb; 6: hypothalamus.


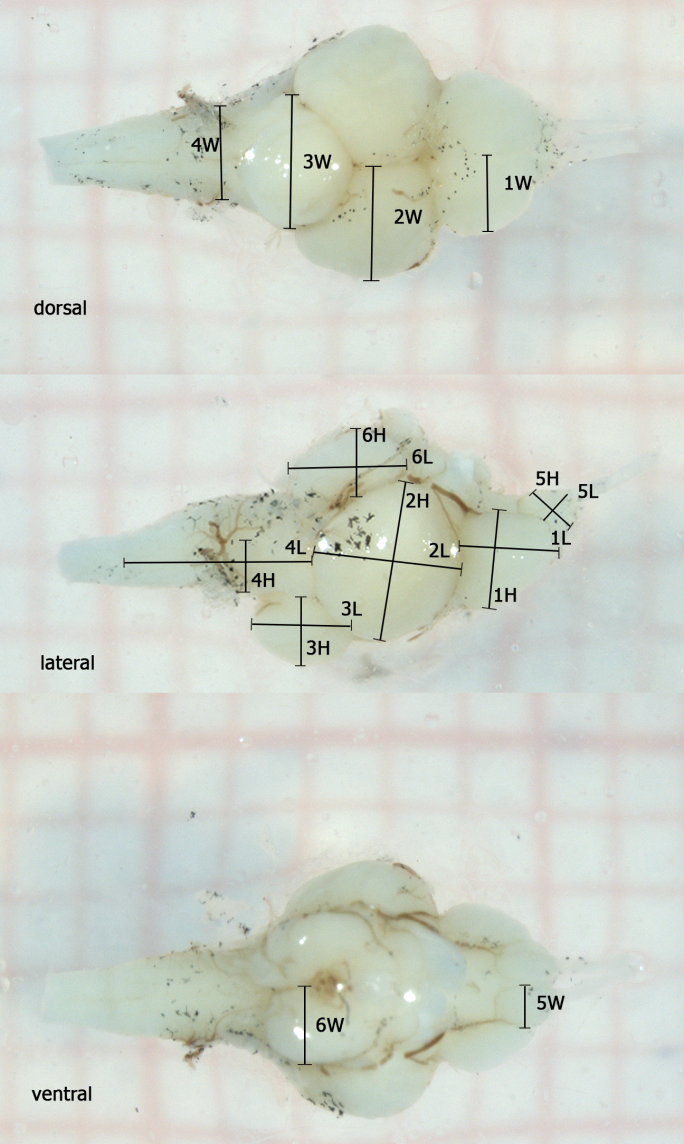

Supplement: Supplementary file 1 [file ECE3-7-1691-s001.docx]
