# Supplementary material for: Environmental enrichment, sexual dimorphism, and brain size in sticklebacks
Source: Ecol Evol. 2017 Feb 12;7(6):1691–8. doi: 10.1002/ece3.2717 (PMC5355184; doi:10.1002/ece3.2717)
Supplement: Supplementary file 2 [file ECE3-7-1691-s002.docx]

**Appendix S2.** Means and 95% confidence intervals for different brain and body traits in Control and Treatment groups conditioned for fixed and random effects in Table 2. The effect sizes (Cohen’s *d*) for treatment effects are also included, which all indicate very weak treatment effects. Hence, the lack of significant treatment effects is unlikely due to poor power for detecting meaningful effect, but rather, to small effect size. Note that *d* estimates are conservative in the sense that they were calculated assuming n = 8 per treatment group: use of larger n would have made them even smaller.

Treatment

Trait Control Treatment Effect size (*d*)

Mean 95%CI Mean 95%CI

Brain size (g× 10^2^) 1.32 1.29 – 1.35 1.31 1.28 – 1.34 0.19

Brain size (mm^3^) 9.33 9.06 – 9.63 9.30 9.01 – 9.63 0.07

Dorsal medulla (mm^3^) 0.66 0.62 – 0.69 0.67 0.63 – 0.70 -0.19

Telencephalon (mm^3^) 1.66 1.59 – 1.63 1.66 1.58 – 1.74 0.02

Optic tectum (mm^3^) 5.19 5.00 – 5.39 5.24 5.03 – 5.44 -0.15

Cerebellum (mm^3^) 0.81 0.77 – 0.84 0.78 0.76 – 0.82 0.44

Olfactory bulb (mm^3^× 10^2^) 1.21 1.10 – 1.32 1.21 1.10 – 1.33 -0.02

Hypothalamus (mm^3^) 1.43 1.29 – 1.58 1.46 1.32 – 1.62 -0.14

Standard length (mm) 37.83 37.83 – 38.37 38.02 37.47 – 38.39 -0.23

Body mass (g) 0.70 0.64 – 0.76 0.73 0.67 – 0.79 -0.34

Condition (g) 0.70 0.66 – 0.70 0.72 0.68 –0.76 0.32
